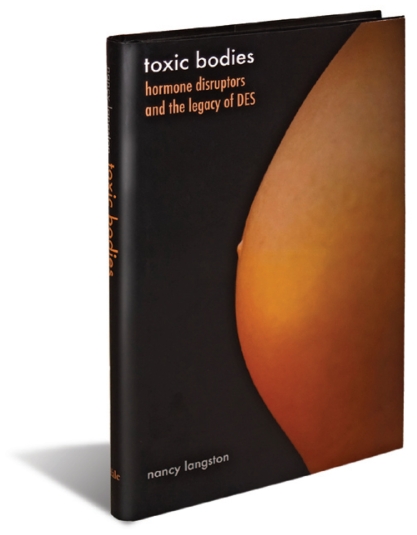# Toxic Bodies: Hormone Disruptors and the Legacy of DES

**Published:** 2010-10

**Authors:** Retha R. Newbold

**Affiliations:** Retha R. Newbold is a developmental reproductive biologist working at the National Institute of Environmental Health Sciences for 37 years until retirement in 2009. Early in her career, she developed a mouse model to study prenatal effects of DES, which proved useful in replicating and predicting adverse effects in humans. She continues to use this model to study other endocrine disrupting chemicals. Newbold is the author of more than 200 publications in the field

Nancy Langston examines the history of synthetic chemicals that disrupt hormones in our bodies and the ecosystem, and the struggles government agencies have faced in regulating them. She uses the powerful case study of the synthetic estrogen diethylstilbestrol (DES) as an example of what can go wrong if precautionary measures are not followed in protecting public health. Although DES was ultimately prescribed for use in all pregnancies to ensure bigger, healthier babies, there was no science that supported safe use in normal pregnancy. She helps answer the question of why DES was so widely used in the face of conflicting evidence over its efficacy and safety. Langston presents a well-documented historical account of DES and parallels its story with the indiscriminate use of numerous other endocrine-disrupting chemicals today. She points out the disturbing possibility of a repeat of the DES scenario.

DES was first synthesized in 1938 by British biochemist Sir Edward Charles Dodds who, along with colleagues, searched for synthetic estrogens that could be easily and inexpensively made to treat symptoms of menopause, termed a “deficiency disease” by the medical profession. By 1941, DES was found in almost every aspect of American life, although researchers knew that it caused cancer and sexual dysfunction in experimental animals and their offspring. Not only was it widely prescribed as hormone replacement therapy, it was also given to pregnant women at risk for miscarriage. DES became so popular that it was prescribed for normal healthy pregnancies much like vitamin pills, although it was never demonstrated to have beneficial effects, and many women were never informed they were given the drug. DES was also used to treat numerous other medical conditions including breast and prostate cancer, stop production of milk after childbirth for non-nursing women, treat menstrual disorders, treat acne, and halt the growth of young girls considered at risk for getting too tall. In addition, DES was quickly introduced into veterinary practice to treat infertility and mastitis in livestock, as pellets implanted into poultry and livestock and as feed additives for cattle to improve feed efficiency and promote rapid weight gain. Langston cites a report stating that, by 1955, > 90% of the livestock in this country was being given DES.

Today we know the harmful effects of DES, especially if exposure occurs during prenatal development; at pharmaceutical levels, there is a low but significant increase (< 0.01%) in cancer in young women who were exposed before birth, and there is a high incidence (> 90%) of non-cancer reproductive abnormalities in both male and female offspring. The full extent of DES’s adverse effects remains to be determined as the exposed population ages, but it may involve additional generations (DES grandchildren). Describing low levels of DES exposure such as those obtained from eating meat, Langston compellingly argues that DES and other chemical residues are playing a role in current health problems such as increased infertility and cancer rates.

Almost immediately after its introduction to the public, DES posed problems for regulatory agencies charged with protecting public health while not stifling the marketing of beneficial drugs. DES came before the Food and Drug Administration (FDA) three different times, and despite the agency’s concern for safety, it backed way from regulatory actions due to strong political pressure from the pharmaceutical and agricultural communities. Langston does not fault FDA or other government scientists for failure to act, but rather points to many dedicated investigators and concerned politicians who worked in the past to protect human health. She does, however, unapologetically name some early government administrators, politicians, and industry representatives who, as she states, “chose the health of big business over public good, and . . . skillfully manipulated scientific uncertainty to delay regulation.” She points out that the old FDA mind-set to protect industry and promote the economy is still entrenched in the FDA today.

As an environmental historian, Langston sees a similar pattern in the lack of regulation between DES in the past and endocrine-disrupting chemicals such as bisphenol A today. The purpose of Langston’s book is to point out how lessons from history can be used to make better policy, as well as “the need for intelligent regulation to protect public health and the environment.” She challenges readers to stay informed, take action, and support government agencies in their struggle in applying the precautionary principle to protect human health. She convincingly advocates that public health concerns should take precedence over short-term economic gains in the face of scientific uncertainty.

## Figures and Tables

**Figure:**